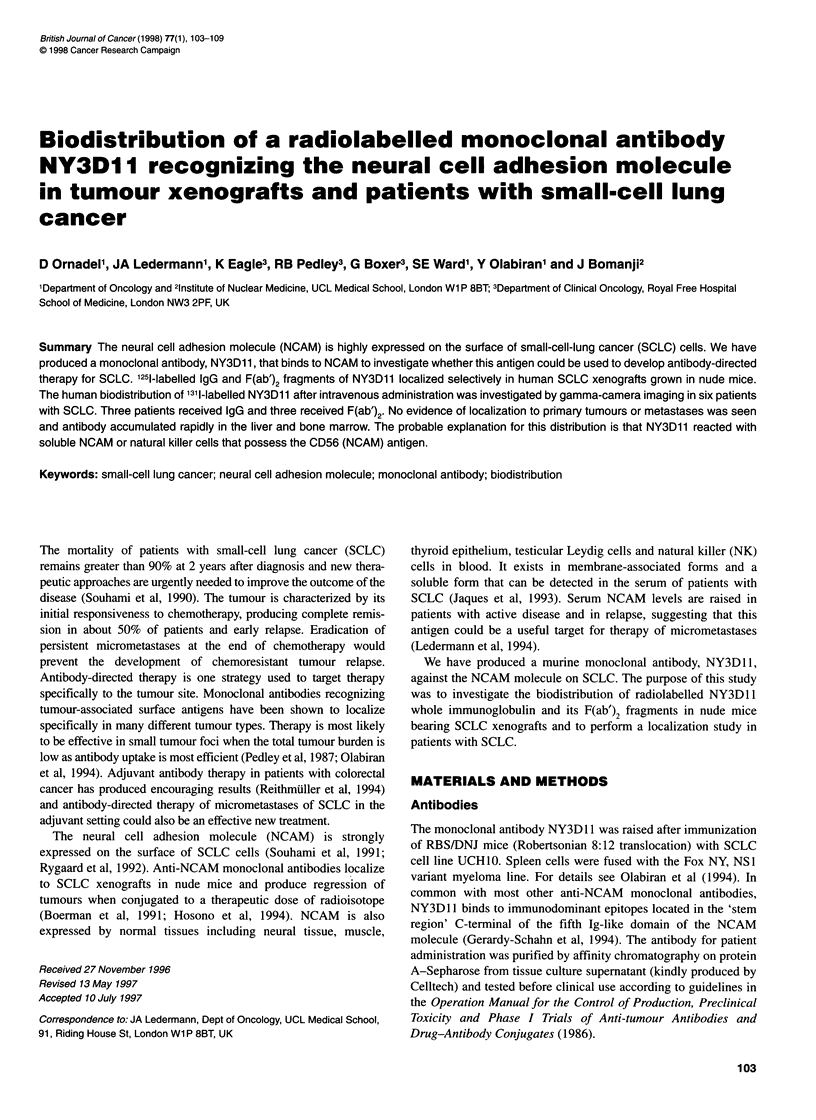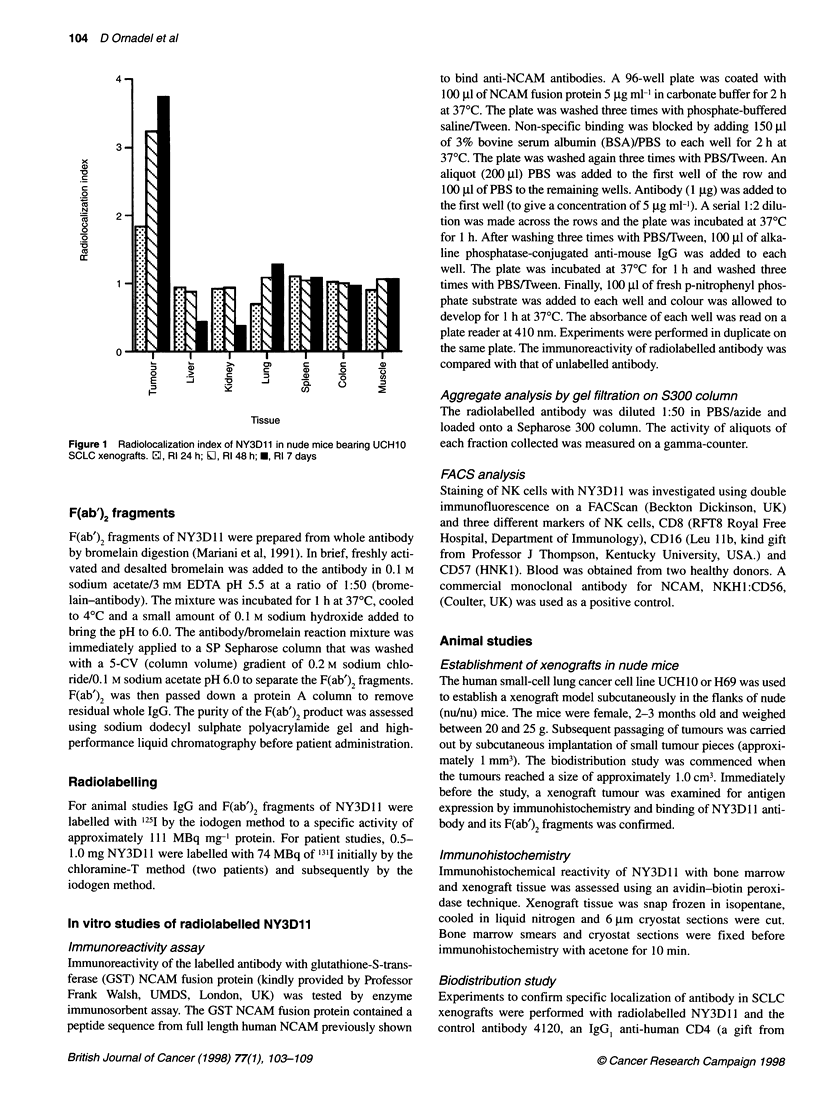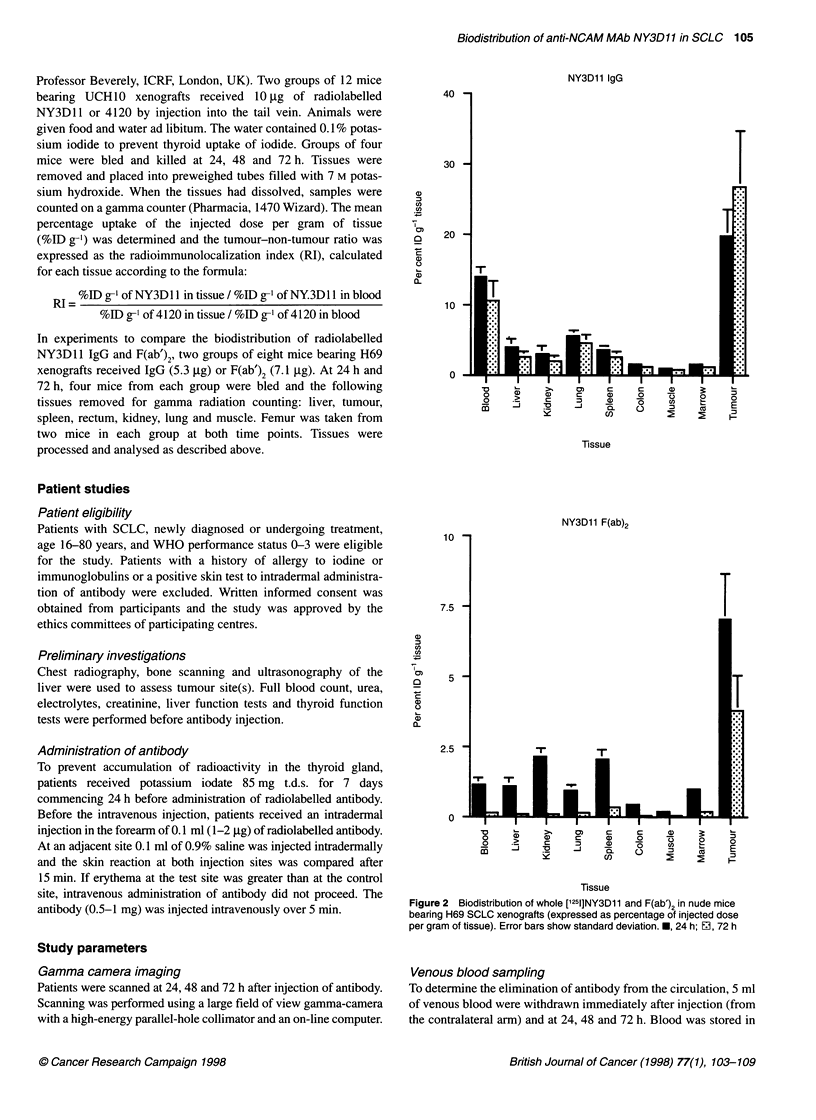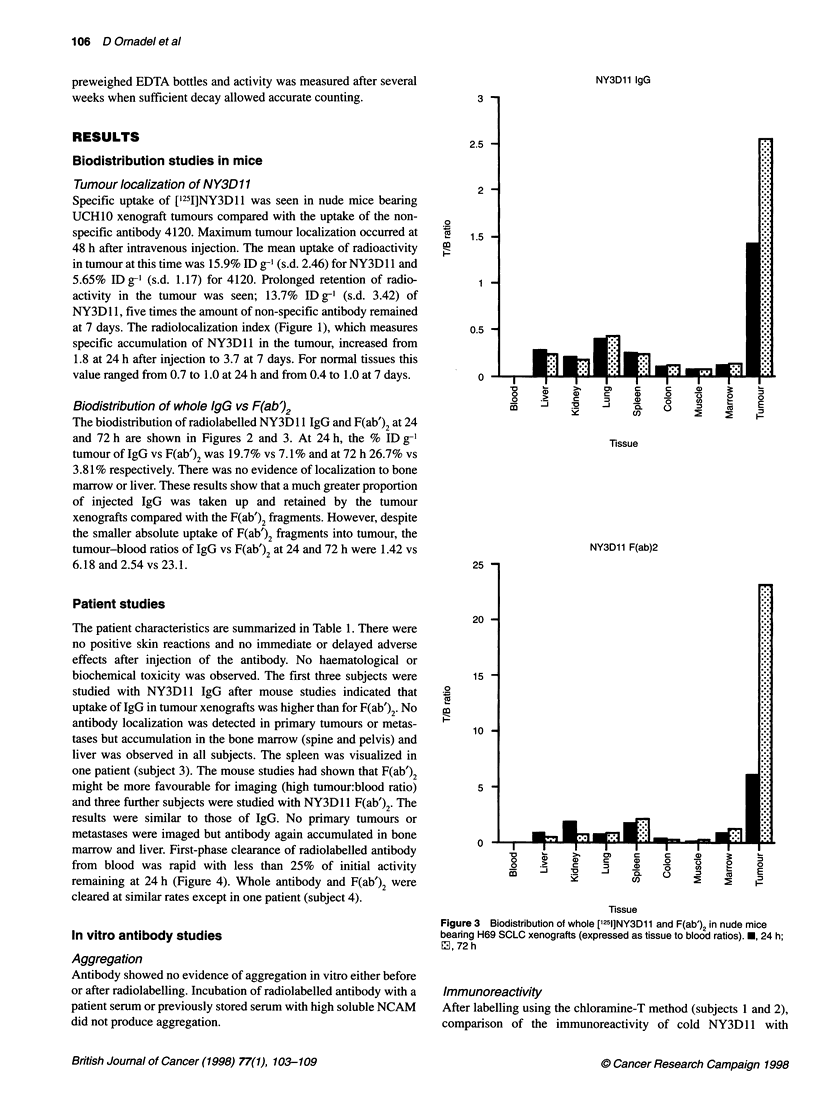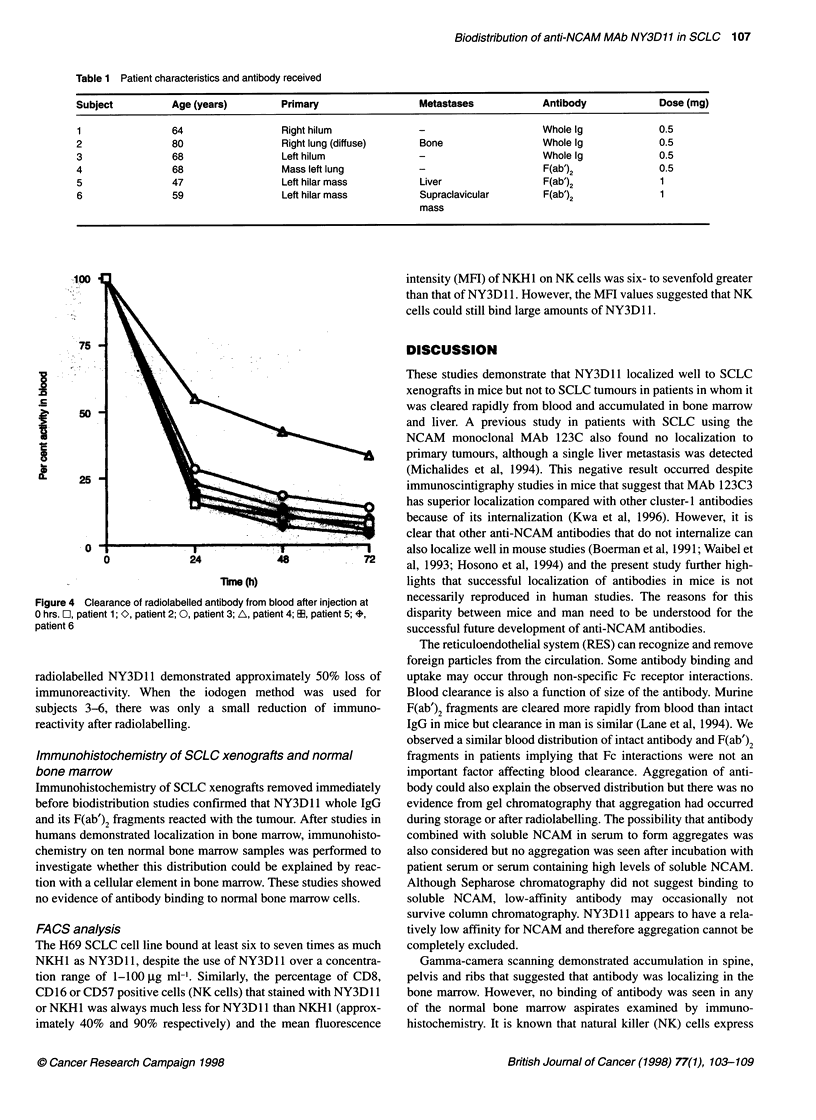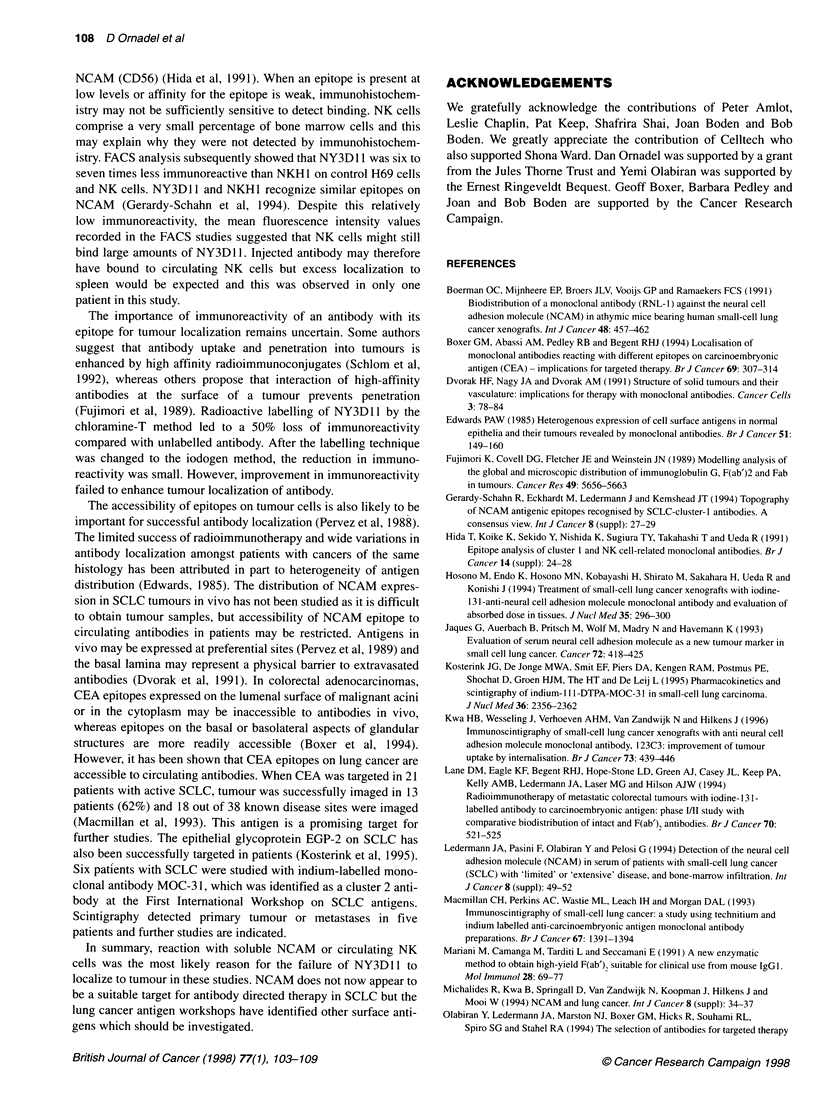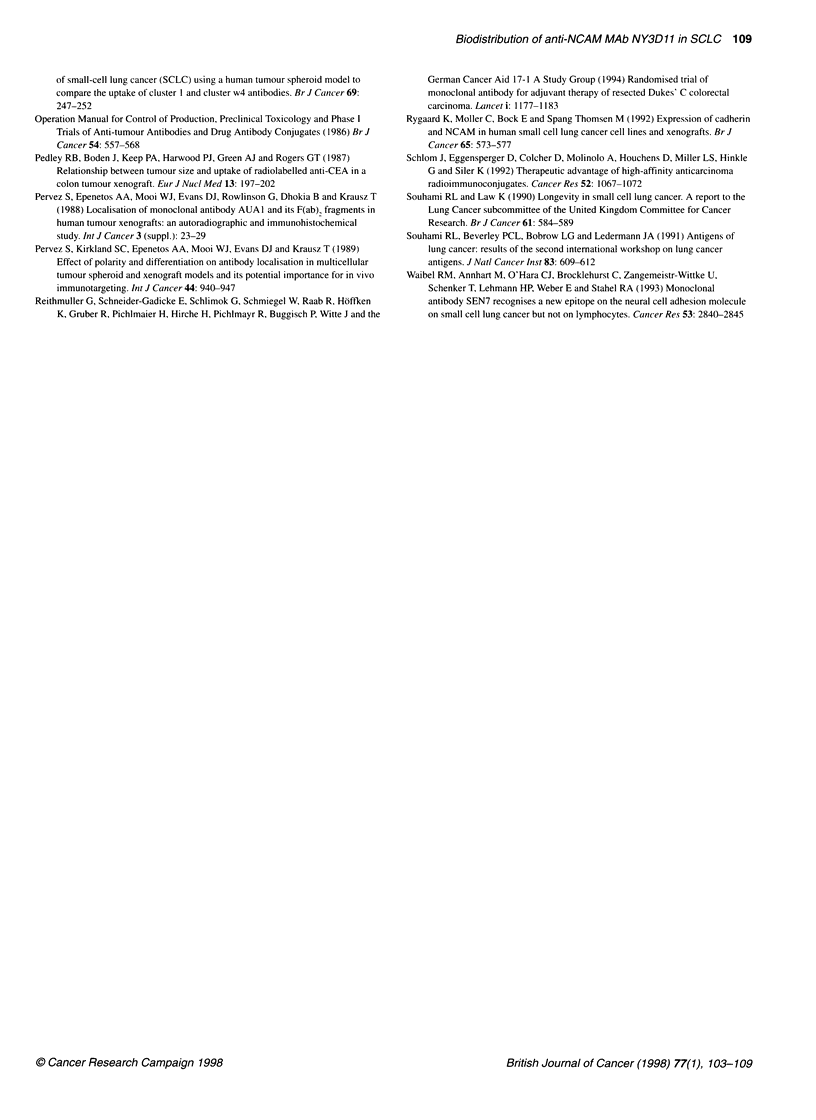# Biodistribution of a radiolabelled monoclonal antibody NY3D11 recognizing the neural cell adhesion molecule in tumour xenografts and patients with small-cell lung cancer.

**DOI:** 10.1038/bjc.1998.16

**Published:** 1998

**Authors:** D. Ornadel, J. A. Ledermann, K. Eagle, R. B. Pedley, G. Boxer, S. E. Ward, Y. Olabiran, J. Bomanji

**Affiliations:** Department of Oncology, UCL Medical School, London, UK.

## Abstract

The neural cell adhesion molecule (NCAM) is highly expressed on the surface of small-cell-lung cancer (SCLC) cells. We have produced a monoclonal antibody, NY3D11, that binds to NCAM to investigate whether this antigen could be used to develop antibody-directed therapy for SCLC. 125I-labelled IgG and F(ab')2 fragments of NY3D11 localized selectively in human SCLC xenografts grown in nude mice. The human biodistribution of 131I-labelled NY3D11 after intravenous administration was investigated by gamma-camera imaging in six patients with SCLC. Three patients received IgG and three received F(ab')2. No evidence of localization to primary tumours or metastases was seen and antibody accumulated rapidly in the liver and bone marrow. The probable explanation for this distribution is that NY3D11 reacted with soluble NCAM or natural killer cells that possess the CD56 (NCAM) antigen.


British Journal of Cancer (1998) 77(1), 103–109
© 1998 Cancer Research Campaign

### Biodistribution of a radiolabelled monoclonal antibody NY3D11 recognizing the neural cell adhesion molecule in tumour xenografts and patients with small-cell lung cancer

**D Ornadel[1], JA Ledermann[1], K Eagle[3], RB Pedley[3], G Boxer[3], SE Ward[1], Y Olabiran[1] and J Bomanji[2]**

[1]Department of Oncology and [2]Institute of Nuclear Medicine, UCL Medical School, London W1P 8BT; [3]Department of Clinical Oncology, Royal Free Hospital School of Medicine, London NW3 2PF, UK

**Summary** The neural cell adhesion molecule (NCAM) is highly expressed on the surface of small-cell-lung cancer (SCLC) cells. We have produced a monoclonal antibody, NY3D11, that binds to NCAM to investigate whether this antigen could be used to develop antibody-directed therapy for SCLC. $^{125}$I-labelled IgG and F(ab′)$_2$ fragments of NY3D11 localized selectively in human SCLC xenografts grown in nude mice. The human biodistribution of $^{131}$I-labelled NY3D11 after intravenous administration was investigated by gamma-camera imaging in six patients with SCLC. Three patients received IgG and three received F(ab′)$_2$. No evidence of localization to primary tumours or metastases was seen and antibody accumulated rapidly in the liver and bone marrow. The probable explanation for this distribution is that NY3D11 reacted with soluble NCAM or natural killer cells that possess the CD56 (NCAM) antigen.

**Keywords:** small-cell lung cancer; neural cell adhesion molecule; monoclonal antibody; biodistribution

The mortality of patients with small-cell lung cancer (SCLC) remains greater than 90% at 2 years after diagnosis and new therapeutic approaches are urgently needed to improve the outcome of the disease (Souhami et al, 1990). The tumour is characterized by its initial responsiveness to chemotherapy, producing complete remission in about 50% of patients and early relapse. Eradication of persistent micrometastases at the end of chemotherapy would prevent the development of chemoresistant tumour relapse. Antibody-directed therapy is one strategy used to target therapy specifically to the tumour site. Monoclonal antibodies recognizing tumour-associated surface antigens have been shown to localize specifically in many different tumour types. Therapy is most likely to be effective in small tumour foci when the total tumour burden is low as antibody uptake is most efficient (Pedley et al, 1987; Olabiran et al, 1994). Adjuvant antibody therapy in patients with colorectal cancer has produced encouraging results (Reithmüller et al, 1994) and antibody-directed therapy of micrometastases of SCLC in the adjuvant setting could also be an effective new treatment.

The neural cell adhesion molecule (NCAM) is strongly expressed on the surface of SCLC cells (Souhami et al, 1991; Rygaard et al, 1992). Anti-NCAM monoclonal antibodies localize to SCLC xenografts in nude mice and produce regression of tumours when conjugated to a therapeutic dose of radioisotope (Boerman et al, 1991; Hosono et al, 1994). NCAM is also expressed by normal tissues including neural tissue, muscle, thyroid epithelium, testicular Leydig cells and natural killer (NK) cells in blood. It exists in membrane-associated forms and a soluble form that can be detected in the serum of patients with SCLC (Jaques et al, 1993). Serum NCAM levels are raised in patients with active disease and in relapse, suggesting that this antigen could be a useful target for therapy of micrometastases (Ledermann et al, 1994).

We have produced a murine monoclonal antibody, NY3D11, against the NCAM molecule on SCLC. The purpose of this study was to investigate the biodistribution of radiolabelled NY3D11 whole immunoglobulin and its F(ab′)$_2$ fragments in nude mice bearing SCLC xenografts and to perform a localization study in patients with SCLC.

*Received 27 November 1996*
*Revised 13 May 1997*
*Accepted 10 July 1997*

*Correspondence to:* JA Ledermann, Dept of Oncology, UCL Medical School, 91, Riding House St, London W1P 8BT, UK

### MATERIALS AND METHODS

### Antibodies

The monoclonal antibody NY3D11 was raised after immunization of RBS/DNJ mice (Robertsonian 8:12 translocation) with SCLC cell line UCH10. Spleen cells were fused with the Fox NY, NS1 variant myeloma line. For details see Olabiran et al (1994). In common with most other anti-NCAM monoclonal antibodies, NY3D11 binds to immunodominant epitopes located in the 'stem region' C-terminal of the fifth Ig-like domain of the NCAM molecule (Gerardy-Schahn et al, 1994). The antibody for patient administration was purified by affinity chromatography on protein A–Sepharose from tissue culture supernatant (kindly produced by Celltech) and tested before clinical use according to guidelines in the *Operation Manual for the Control of Production, Preclinical Toxicity and Phase I Trials of Anti-tumour Antibodies and Drug–Antibody Conjugates* (1986).

**Figure 1** Radiolocalization index of NY3D11 in nude mice bearing UCH10 SCLC xenografts. ▒, RI 24 h; ▨, RI 48 h; ■, RI 7 days

### $F(ab')_2$ fragments

$F(ab')_2$ fragments of NY3D11 were prepared from whole antibody by bromelain digestion (Mariani et al, 1991). In brief, freshly activated and desalted bromelain was added to the antibody in 0.1 M sodium acetate/3 mM EDTA pH 5.5 at a ratio of 1:50 (bromelain–antibody). The mixture was incubated for 1 h at 37°C, cooled to 4°C and a small amount of 0.1 M sodium hydroxide added to bring the pH to 6.0. The antibody/bromelain reaction mixture was immediately applied to a SP Sepharose column that was washed with a 5-CV (column volume) gradient of 0.2 M sodium chloride/0.1 M sodium acetate pH 6.0 to separate the $F(ab')_2$ fragments. $F(ab')_2$ was then passed down a protein A column to remove residual whole IgG. The purity of the $F(ab')_2$ product was assessed using sodium dodecyl sulphate polyacrylamide gel and high-performance liquid chromatography before patient administration.

### Radiolabelling

For animal studies IgG and $F(ab')_2$ fragments of NY3D11 were labelled with $^{125}I$ by the iodogen method to a specific activity of approximately 111 MBq $mg^{-1}$ protein. For patient studies, 0.5–1.0 mg NY3D11 were labelled with 74 MBq of $^{131}I$ initially by the chloramine-T method (two patients) and subsequently by the iodogen method.

### In vitro studies of radiolabelled NY3D11

### *Immunoreactivity assay*

Immunoreactivity of the labelled antibody with glutathione-S-transferase (GST) NCAM fusion protein (kindly provided by Professor Frank Walsh, UMDS, London, UK) was tested by enzyme immunosorbent assay. The GST NCAM fusion protein contained a peptide sequence from full length human NCAM previously shown to bind anti-NCAM antibodies. A 96-well plate was coated with 100 μl of NCAM fusion protein 5 μg $ml^{-1}$ in carbonate buffer for 2 h at 37°C. The plate was washed three times with phosphate-buffered saline/Tween. Non-specific binding was blocked by adding 150 μl of 3% bovine serum albumin (BSA)/PBS to each well for 2 h at 37°C. The plate was washed again three times with PBS/Tween. An aliquot (200 μl) PBS was added to the first well of the row and 100 μl of PBS to the remaining wells. Antibody (1 μg) was added to the first well (to give a concentration of 5 μg $ml^{-1}$). A serial 1:2 dilution was made across the rows and the plate was incubated at 37°C for 1 h. After washing three times with PBS/Tween, 100 μl of alkaline phosphatase-conjugated anti-mouse IgG was added to each well. The plate was incubated at 37°C for 1 h and washed three times with PBS/Tween. Finally, 100 μl of fresh p-nitrophenyl phosphate substrate was added to each well and colour was allowed to develop for 1 h at 37°C. The absorbance of each well was read on a plate reader at 410 nm. Experiments were performed in duplicate on the same plate. The immunoreactivity of radiolabelled antibody was compared with that of unlabelled antibody.

### *Aggregate analysis by gel filtration on S300 column*

The radiolabelled antibody was diluted 1:50 in PBS/azide and loaded onto a Sepharose 300 column. The activity of aliquots of each fraction collected was measured on a gamma-counter.

### *FACS analysis*

Staining of NK cells with NY3D11 was investigated using double immunofluorescence on a FACScan (Beckton Dickinson, UK) and three different markers of NK cells, CD8 (RFT8 Royal Free Hospital, Department of Immunology), CD16 (Leu 11b, kind gift from Professor J Thompson, Kentucky University, USA.) and CD57 (HNK1). Blood was obtained from two healthy donors. A commercial monoclonal antibody for NCAM, NKH1:CD56, (Coulter, UK) was used as a positive control.

### Animal studies

### *Establishment of xenografts in nude mice*

The human small-cell lung cancer cell line UCH10 or H69 was used to establish a xenograft model subcutaneously in the flanks of nude (nu/nu) mice. The mice were female, 2–3 months old and weighed between 20 and 25 g. Subsequent passaging of tumours was carried out by subcutaneous implantation of small tumour pieces (approximately 1 $mm^3$). The biodistribution study was commenced when the tumours reached a size of approximately 1.0 $cm^3$. Immediately before the study, a xenograft tumour was examined for antigen expression by immunohistochemistry and binding of NY3D11 antibody and its $F(ab')_2$ fragments was confirmed.

### *Immunohistochemistry*

Immunohistochemical reactivity of NY3D11 with bone marrow and xenograft tissue was assessed using an avidin–biotin peroxidase technique. Xenograft tissue was snap frozen in isopentane, cooled in liquid nitrogen and 6 μm cryostat sections were cut. Bone marrow smears and cryostat sections were fixed before immunohistochemistry with acetone for 10 min.

### *Biodistribution study*

Experiments to confirm specific localization of antibody in SCLC xenografts were performed with radiolabelled NY3D11 and the control antibody 4120, an $IgG_1$ anti-human CD4 (a gift from

© Cancer Research Campaign 1998

Professor Beverely, ICRF, London, UK). Two groups of 12 mice bearing UCH10 xenografts received 10 µg of radiolabelled NY3D11 or 4120 by injection into the tail vein. Animals were given food and water ad libitum. The water contained 0.1% potassium iodide to prevent thyroid uptake of iodide. Groups of four mice were bled and killed at 24, 48 and 72 h. Tissues were removed and placed into preweighed tubes filled with 7 M potassium hydroxide. When the tissues had dissolved, samples were counted on a gamma counter (Pharmacia, 1470 Wizard). The mean percentage uptake of the injected dose per gram of tissue (%ID $g^{-1}$) was determined and the tumour–non-tumour ratio was expressed as the radioimmunolocalization index (RI), calculated for each tissue according to the formula:

$$RI = \frac{\%ID\ g^{-1} \text{ of NY3D11 in tissue} / \%ID\ g^{-1} \text{ of NY.3D11 in blood}}{\%ID\ g^{-1} \text{ of 4120 in tissue} / \%ID\ g^{-1} \text{ of 4120 in blood}}$$

In experiments to compare the biodistribution of radiolabelled NY3D11 IgG and $F(ab')_2$, two groups of eight mice bearing H69 xenografts received IgG (5.3 µg) or $F(ab')_2$ (7.1 µg). At 24 h and 72 h, four mice from each group were bled and the following tissues removed for gamma radiation counting: liver, tumour, spleen, rectum, kidney, lung and muscle. Femur was taken from two mice in each group at both time points. Tissues were processed and analysed as described above.

### Patient studies

### *Patient eligibility*

Patients with SCLC, newly diagnosed or undergoing treatment, age 16–80 years, and WHO performance status 0–3 were eligible for the study. Patients with a history of allergy to iodine or immunoglobulins or a positive skin test to intradermal administration of antibody were excluded. Written informed consent was obtained from participants and the study was approved by the ethics committees of participating centres.

### *Preliminary investigations*

Chest radiography, bone scanning and ultrasonography of the liver were used to assess tumour site(s). Full blood count, urea, electrolytes, creatinine, liver function tests and thyroid function tests were performed before antibody injection.

### *Administration of antibody*

To prevent accumulation of radioactivity in the thyroid gland, patients received potassium iodate 85 mg t.d.s. for 7 days commencing 24 h before administration of radiolabelled antibody. Before the intravenous injection, patients received an intradermal injection in the forearm of 0.1 ml (1–2 µg) of radiolabelled antibody. At an adjacent site 0.1 ml of 0.9% saline was injected intradermally and the skin reaction at both injection sites was compared after 15 min. If erythema at the test site was greater than at the control site, intravenous administration of antibody did not proceed. The antibody (0.5–1 mg) was injected intravenously over 5 min.

### Study parameters

### *Gamma camera imaging*

Patients were scanned at 24, 48 and 72 h after injection of antibody. Scanning was performed using a large field of view gamma-camera with a high-energy parallel-hole collimator and an on-line computer.

**Figure 2** Biodistribution of whole [$^{125}$I]NY3D11 and $F(ab')_2$ in nude mice bearing H69 SCLC xenografts (expressed as percentage of injected dose per gram of tissue). Error bars show standard deviation. ■, 24 h; ▨, 72 h

### *Venous blood sampling*

To determine the elimination of antibody from the circulation, 5 ml of venous blood were withdrawn immediately after injection (from the contralateral arm) and at 24, 48 and 72 h. Blood was stored in

© Cancer Research Campaign 1998

preweighed EDTA bottles and activity was measured after several weeks when sufficient decay allowed accurate counting.

### RESULTS

### Biodistribution studies in mice

#### *Tumour localization of NY3D11*

Specific uptake of [$^{125}$I]NY3D11 was seen in nude mice bearing UCH10 xenograft tumours compared with the uptake of the non-specific antibody 4120. Maximum tumour localization occurred at 48 h after intravenous injection. The mean uptake of radioactivity in tumour at this time was 15.9% ID g$^{-1}$ (s.d. 2.46) for NY3D11 and 5.65% ID g$^{-1}$ (s.d. 1.17) for 4120. Prolonged retention of radioactivity in the tumour was seen; 13.7% ID g$^{-1}$ (s.d. 3.42) of NY3D11, five times the amount of non-specific antibody remained at 7 days. The radiolocalization index (Figure 1), which measures specific accumulation of NY3D11 in the tumour, increased from 1.8 at 24 h after injection to 3.7 at 7 days. For normal tissues this value ranged from 0.7 to 1.0 at 24 h and from 0.4 to 1.0 at 7 days.

#### *Biodistribution of whole IgG vs F(ab′)$_2$*

The biodistribution of radiolabelled NY3D11 IgG and F(ab′)$_2$ at 24 and 72 h are shown in Figures 2 and 3. At 24 h, the % ID g$^{-1}$ tumour of IgG vs F(ab′)$_2$ was 19.7% vs 7.1% and at 72 h 26.7% vs 3.81% respectively. There was no evidence of localization to bone marrow or liver. These results show that a much greater proportion of injected IgG was taken up and retained by the tumour xenografts compared with the F(ab′)$_2$ fragments. However, despite the smaller absolute uptake of F(ab′)$_2$ fragments into tumour, the tumour–blood ratios of IgG vs F(ab′)$_2$ at 24 and 72 h were 1.42 vs 6.18 and 2.54 vs 23.1.

### Patient studies

The patient characteristics are summarized in Table 1. There were no positive skin reactions and no immediate or delayed adverse effects after injection of the antibody. No haematological or biochemical toxicity was observed. The first three subjects were studied with NY3D11 IgG after mouse studies indicated that uptake of IgG in tumour xenografts was higher than for F(ab′)$_2$. No antibody localization was detected in primary tumours or metastases but accumulation in the bone marrow (spine and pelvis) and liver was observed in all subjects. The spleen was visualized in one patient (subject 3). The mouse studies had shown that F(ab′)$_2$ might be more favourable for imaging (high tumour:blood ratio) and three further subjects were studied with NY3D11 F(ab′)$_2$. The results were similar to those of IgG. No primary tumours or metastases were imaged but antibody again accumulated in bone marrow and liver. First-phase clearance of radiolabelled antibody from blood was rapid with less than 25% of initial activity remaining at 24 h (Figure 4). Whole antibody and F(ab′)$_2$ were cleared at similar rates except in one patient (subject 4).

### In vitro antibody studies

#### *Aggregation*

Antibody showed no evidence of aggregation in vitro either before or after radiolabelling. Incubation of radiolabelled antibody with a patient serum or previously stored serum with high soluble NCAM did not produce aggregation.

**Figure 3** Biodistribution of whole [$^{125}$I]NY3D11 and F(ab′)$_2$ in nude mice bearing H69 SCLC xenografts (expressed as tissue to blood ratios). ■, 24 h; ⚃, 72 h

#### *Immunoreactivity*

After labelling using the chloramine-T method (subjects 1 and 2), comparison of the immunoreactivity of cold NY3D11 with

© *Cancer Research Campaign 1998*

**Table 1** Patient characteristics and antibody received

| Subject | Age (years) | Primary | Metastases | Antibody | Dose (mg) |
|---|---|---|---|---|---|
| 1 | 64 | Right hilum | – | Whole Ig | 0.5 |
| 2 | 80 | Right lung (diffuse) | Bone | Whole Ig | 0.5 |
| 3 | 68 | Left hilum | – | Whole Ig | 0.5 |
| 4 | 68 | Mass left lung | – | F(ab')$_2$ | 0.5 |
| 5 | 47 | Left hilar mass | Liver | F(ab')$_2$ | 1 |
| 6 | 59 | Left hilar mass | Supraclavicular mass | F(ab')$_2$ | 1 |

**Figure 4** Clearance of radiolabelled antibody from blood after injection at 0 hrs. □, patient 1; ◇, patient 2; ○, patient 3; △, patient 4; ⊞, patient 5; ⊕, patient 6

radiolabelled NY3D11 demonstrated approximately 50% loss of immunoreactivity. When the iodogen method was used for subjects 3–6, there was only a small reduction of immunoreactivity after radiolabelling.

### *Immunohistochemistry of SCLC xenografts and normal bone marrow*

Immunohistochemistry of SCLC xenografts removed immediately before biodistribution studies confirmed that NY3D11 whole IgG and its F(ab')$_2$ fragments reacted with the tumour. After studies in humans demonstrated localization in bone marrow, immunohistochemistry on ten normal bone marrow samples was performed to investigate whether this distribution could be explained by reaction with a cellular element in bone marrow. These studies showed no evidence of antibody binding to normal bone marrow cells.

### *FACS analysis*

The H69 SCLC cell line bound at least six to seven times as much NKH1 as NY3D11, despite the use of NY3D11 over a concentration range of 1–100 μg ml$^{-1}$. Similarly, the percentage of CD8, CD16 or CD57 positive cells (NK cells) that stained with NY3D11 or NKH1 was always much less for NY3D11 than NKH1 (approximately 40% and 90% respectively) and the mean fluorescence intensity (MFI) of NKH1 on NK cells was six- to sevenfold greater than that of NY3D11. However, the MFI values suggested that NK cells could still bind large amounts of NY3D11.

### DISCUSSION

These studies demonstrate that NY3D11 localized well to SCLC xenografts in mice but not to SCLC tumours in patients in whom it was cleared rapidly from blood and accumulated in bone marrow and liver. A previous study in patients with SCLC using the NCAM monoclonal MAb 123C also found no localization to primary tumours, although a single liver metastasis was detected (Michalides et al, 1994). This negative result occurred despite immunoscintigraphy studies in mice that suggest that MAb 123C3 has superior localization compared with other cluster-1 antibodies because of its internalization (Kwa et al, 1996). However, it is clear that other anti-NCAM antibodies that do not internalize can also localize well in mouse studies (Boerman et al, 1991; Waibel et al, 1993; Hosono et al, 1994) and the present study further highlights that successful localization of antibodies in mice is not necessarily reproduced in human studies. The reasons for this disparity between mice and man need to be understood for the successful future development of anti-NCAM antibodies.

The reticuloendothelial system (RES) can recognize and remove foreign particles from the circulation. Some antibody binding and uptake may occur through non-specific Fc receptor interactions. Blood clearance is also a function of size of the antibody. Murine F(ab')$_2$ fragments are cleared more rapidly from blood than intact IgG in mice but clearance in man is similar (Lane et al, 1994). We observed a similar blood distribution of intact antibody and F(ab')$_2$ fragments in patients implying that Fc interactions were not an important factor affecting blood clearance. Aggregation of antibody could also explain the observed distribution but there was no evidence from gel chromatography that aggregation had occurred during storage or after radiolabelling. The possibility that antibody combined with soluble NCAM in serum to form aggregates was also considered but no aggregation was seen after incubation with patient serum or serum containing high levels of soluble NCAM. Although Sepharose chromatography did not suggest binding to soluble NCAM, low-affinity antibody may occasionally not survive column chromatography. NY3D11 appears to have a relatively low affinity for NCAM and therefore aggregation cannot be completely excluded.

Gamma-camera scanning demonstrated accumulation in spine, pelvis and ribs that suggested that antibody was localizing in the bone marrow. However, no binding of antibody was seen in any of the normal bone marrow aspirates examined by immunohistochemistry. It is known that natural killer (NK) cells express

© Cancer Research Campaign 1998

NCAM (CD56) (Hida et al, 1991). When an epitope is present at low levels or affinity for the epitope is weak, immunohistochemistry may not be sufficiently sensitive to detect binding. NK cells comprise a very small percentage of bone marrow cells and this may explain why they were not detected by immunohistochemistry. FACS analysis subsequently showed that NY3D11 was six to seven times less immunoreactive than NKH1 on control H69 cells and NK cells. NY3D11 and NKH1 recognize similar epitopes on NCAM (Gerardy-Schahn et al, 1994). Despite this relatively low immunoreactivity, the mean fluorescence intensity values recorded in the FACS studies suggested that NK cells might still bind large amounts of NY3D11. Injected antibody may therefore have bound to circulating NK cells but excess localization to spleen would be expected and this was observed in only one patient in this study.

The importance of immunoreactivity of an antibody with its epitope for tumour localization remains uncertain. Some authors suggest that antibody uptake and penetration into tumours is enhanced by high affinity radioimmunoconjugates (Schlom et al, 1992), whereas others propose that interaction of high-affinity antibodies at the surface of a tumour prevents penetration (Fujimori et al, 1989). Radioactive labelling of NY3D11 by the chloramine-T method led to a 50% loss of immunoreactivity compared with unlabelled antibody. After the labelling technique was changed to the iodogen method, the reduction in immunoreactivity was small. However, improvement in immunoreactivity failed to enhance tumour localization of antibody.

The accessibility of epitopes on tumour cells is also likely to be important for successful antibody localization (Pervez et al, 1988). The limited success of radioimmunotherapy and wide variations in antibody localization amongst patients with cancers of the same histology has been attributed in part to heterogeneity of antigen distribution (Edwards, 1985). The distribution of NCAM expression in SCLC tumours in vivo has not been studied as it is difficult to obtain tumour samples, but accessibility of NCAM epitope to circulating antibodies in patients may be restricted. Antigens in vivo may be expressed at preferential sites (Pervez et al, 1989) and the basal lamina may represent a physical barrier to extravasated antibodies (Dvorak et al, 1991). In colorectal adenocarcinomas, CEA epitopes expressed on the lumenal surface of malignant acini or in the cytoplasm may be inaccessible to antibodies in vivo, whereas epitopes on the basal or basolateral aspects of glandular structures are more readily accessible (Boxer et al, 1994). However, it has been shown that CEA epitopes on lung cancer are accessible to circulating antibodies. When CEA was targeted in 21 patients with active SCLC, tumour was successfully imaged in 13 patients (62%) and 18 out of 38 known disease sites were imaged (Macmillan et al, 1993). This antigen is a promising target for further studies. The epithelial glycoprotein EGP-2 on SCLC has also been successfully targeted in patients (Kosterink et al, 1995). Six patients with SCLC were studied with indium-labelled monoclonal antibody MOC-31, which was identified as a cluster 2 antibody at the First International Workshop on SCLC antigens. Scintigraphy detected primary tumour or metastases in five patients and further studies are indicated.

In summary, reaction with soluble NCAM or circulating NK cells was the most likely reason for the failure of NY3D11 to localize to tumour in these studies. NCAM does not now appear to be a suitable target for antibody directed therapy in SCLC but the lung cancer antigen workshops have identified other surface antigens which should be investigated.

### ACKNOWLEDGEMENTS

We gratefully acknowledge the contributions of Peter Amlot, Leslie Chaplin, Pat Keep, Shafrira Shai, Joan Boden and Bob Boden. We greatly appreciate the contribution of Celltech who also supported Shona Ward. Dan Ornadel was supported by a grant from the Jules Thorne Trust and Yemi Olabiran was supported by the Ernest Ringeveldt Bequest. Geoff Boxer, Barbara Pedley and Joan and Bob Boden are supported by the Cancer Research Campaign.

### REFERENCES

Boerman OC, Mijnheere EP, Broers JLV, Vooijs GP and Ramaekers FCS (1991) Biodistribution of a monoclonal antibody (RNL-1) against the neural cell adhesion molecule (NCAM) in athymic mice bearing human small-cell lung cancer xenografts. *Int J Cancer* **48**: 457–462

Boxer GM, Abassi AM, Pedley RB and Begent RHJ (1994) Localisation of monoclonal antibodies reacting with different epitopes on carcinoembryonic antigen (CEA) – implications for targeted therapy. *Br J Cancer* **69**: 307–314

Dvorak HF, Nagy JA and Dvorak AM (1991) Structure of solid tumours and their vasculature: implications for therapy with monoclonal antibodies. *Cancer Cells* **3**: 78–84

Edwards PAW (1985) Heterogenous expression of cell surface antigens in normal epithelia and their tumours revealed by monoclonal antibodies. *Br J Cancer* **51**: 149–160

Fujimori K, Covell DG, Fletcher JE and Weinstein JN (1989) Modelling analysis of the global and microscopic distribution of immunoglobulin G, F(ab')2 and Fab in tumours. *Cancer Res* **49**: 5656–5663

Gerardy-Schahn R, Eckhardt M, Ledermann J and Kemshead JT (1994) Topography of NCAM antigenic epitopes recognised by SCLC-cluster-1 antibodies. A consensus view. *Int J Cancer* **8** (suppl): 27–29

Hida T, Koike K, Sekido Y, Nishida K, Sugiura TY, Takahashi T and Ueda R (1991) Epitope analysis of cluster 1 and NK cell-related monoclonal antibodies. *Br J Cancer* **14** (suppl): 24–28

Hosono M, Endo K, Hosono MN, Kobayashi H, Shirato M, Sakahara H, Ueda R and Konishi J (1994) Treatment of small-cell lung cancer xenografts with iodine-131-anti-neural cell adhesion molecule monoclonal antibody and evaluation of absorbed dose in tissues. *J Nucl Med* **35**: 296–300

Jaques G, Auerbach B, Pritsch M, Wolf M, Madry N and Havemann K (1993) Evaluation of serum neural cell adhesion molecule as a new tumour marker in small cell lung cancer. *Cancer* **72**: 418–425

Kosterink JG, De Jonge MWA, Smit EF, Piers DA, Kengen RAM, Postmus PE, Shochat D, Groen HJM, The HT and De Leij L (1995) Pharmacokinetics and scintigraphy of indium-111-DTPA-MOC-31 in small-cell lung carcinoma. *J Nucl Med* **36**: 2356–2362

Kwa HB, Wesseling J, Verhoeven AHM, Van Zandwijk N and Hilkens J (1996) Immunoscintigraphy of small-cell lung cancer xenografts with anti neural cell adhesion molecule monoclonal antibody, 123C3: improvement of tumour uptake by internalisation. *Br J Cancer* **73**: 439–446

Lane DM, Eagle KF, Begent RHJ, Hope-Stone LD, Green AJ, Casey JL, Keep PA, Kelly AMB, Ledermann JA, Laser MG and Hilson AJW (1994) Radioimmunotherapy of metastatic colorectal tumours with iodine-131-labelled antibody to carcinoembryonic antigen: phase I/II study with comparative biodistribution of intact and $F(ab')_2$ antibodies. *Br J Cancer* **70**: 521–525

Ledermann JA, Pasini F, Olabiran Y and Pelosi G (1994) Detection of the neural cell adhesion molecule (NCAM) in serum of patients with small-cell lung cancer (SCLC) with 'limited' or 'extensive' disease, and bone-marrow infiltration. *Int J Cancer* **8** (suppl): 49–52

Macmillan CH, Perkins AC, Wastie ML, Leach IH and Morgan DAL (1993) Immunoscintigraphy of small-cell lung cancer: a study using technitium and indium labelled anti-carcinoembryonic antigen monoclonal antibody preparations. *Br J Cancer* **67**: 1391–1394

Mariani M, Camanga M, Tarditi L and Seccamani E (1991) A new enzymatic method to obtain high-yield $F(ab')_2$ suitable for clinical use from mouse IgG1. *Mol Immunol* **28**: 69–77

Michalides R, Kwa B, Springall D, Van Zandwijk N, Koopman J, Hilkens J and Mooi W (1994) NCAM and lung cancer. *Int J Cancer* **8** (suppl): 34–37

Olabiran Y, Ledermann JA, Marston NJ, Boxer GM, Hicks R, Souhami RL, Spiro SG and Stahel RA (1994) The selection of antibodies for targeted therapy

© Cancer Research Campaign 1998

of small-cell lung cancer (SCLC) using a human tumour spheroid model to compare the uptake of cluster 1 and cluster w4 antibodies. *Br J Cancer* **69**: 247–252

Operation Manual for Control of Production, Preclinical Toxicology and Phase I Trials of Anti-tumour Antibodies and Drug Antibody Conjugates (1986) *Br J Cancer* **54**: 557–568

Pedley RB, Boden J, Keep PA, Harwood PJ, Green AJ and Rogers GT (1987) Relationship between tumour size and uptake of radiolabelled anti-CEA in a colon tumour xenograft. *Eur J Nucl Med* **13**: 197–202

Pervez S, Epenetos AA, Mooi WJ, Evans DJ, Rowlinson G, Dhokia B and Krausz T (1988) Localisation of monoclonal antibody AUA1 and its $F(ab)_2$ fragments in human tumour xenografts: an autoradiographic and immunohistochemical study. *Int J Cancer* **3** (suppl.): 23–29

Pervez S, Kirkland SC, Epenetos AA, Mooi WJ, Evans DJ and Krausz T (1989) Effect of polarity and differentiation on antibody localisation in multicellular tumour spheroid and xenograft models and its potential importance for in vivo immunotargeting. *Int J Cancer* **44**: 940–947

Reithmuller G, Schneider-Gadicke E, Schlimok G, Schmiegel W, Raab R, Höffken K, Gruber R, Pichlmaier H, Hirche H, Pichlmayr R, Buggisch P, Witte J and the German Cancer Aid 17-1 A Study Group (1994) Randomised trial of monoclonal antibody for adjuvant therapy of resected Dukes' C colorectal carcinoma. *Lancet* **i**: 1177–1183

Rygaard K, Moller C, Bock E and Spang Thomsen M (1992) Expression of cadherin and NCAM in human small cell lung cancer cell lines and xenografts. *Br J Cancer* **65**: 573–577

Schlom J, Eggensperger D, Colcher D, Molinolo A, Houchens D, Miller LS, Hinkle G and Siler K (1992) Therapeutic advantage of high-affinity anticarcinoma radioimmunoconjugates. *Cancer Res* **52**: 1067–1072

Souhami RL and Law K (1990) Longevity in small cell lung cancer. A report to the Lung Cancer subcommittee of the United Kingdom Committee for Cancer Research. *Br J Cancer* **61**: 584–589

Souhami RL, Beverley PCL, Bobrow LG and Ledermann JA (1991) Antigens of lung cancer: results of the second international workshop on lung cancer antigens. *J Natl Cancer Inst* **83**: 609–612

Waibel RM, Annhart M, O'Hara CJ, Brocklehurst C, Zangemeistr-Wittke U, Schenker T, Lehmann HP, Weber E and Stahel RA (1993) Monoclonal antibody SEN7 recognises a new epitope on the neural cell adhesion molecule on small cell lung cancer but not on lymphocytes. *Cancer Res* **53**: 2840–2845

© Cancer Research Campaign 1998